# Oltipraz Prevents High Glucose-Induced Oxidative Stress and Apoptosis in RSC96 Cells through the Nrf2/NQO1 Signalling Pathway

**DOI:** 10.1155/2020/5939815

**Published:** 2020-06-23

**Authors:** Zengxin Jiang, Mengxuan Bian, Jingping Wu, Defang Li, Lei Ding, Qingmin Zeng

**Affiliations:** ^1^Department of Orthopedic Surgery, Fudan University Jinshan Hospital, Longhang Road, Jinshan, Shanghai 201508, China; ^2^Department of Orthopedic Surgery, Zhongshan Hospital, Fudan University, Shanghai 200032, China

## Abstract

Diabetic peripheral neuropathy (DPN) is a common complication of diabetes mellitus (DM). Schwann cell (SC) apoptosis contributes to the occurrence and development of DPN. Effective drugs to prevent SC apoptosis are required to relieve and reverse peripheral nerve injury caused by DM. Oltipraz [4-methyl-5-(2-pyrazinyl)-1,2-dithiole-3-thione], an agonist of nuclear factor erythroid derived-2-related factor 2 (Nrf2), exerts strong effect against oxidative stress in animal models or clinical patients in certain diseases, including heart failure, acute kidney injury, and liver injury. The aim of the present study was to determine the effectiveness of oltipraz in preventing SC apoptosis induced by high glucose levels. RSC96 cells pretreated with oltipraz were cultured in high-glucose medium (50 mM glucose) for 24 h, and cells cultured in medium containing 5 mM glucose were used as the control. Flow cytometry was used to evaluate the degree of apoptosis. A Cell Counting Kit-8 assay was used to assess cell viability. The mitochondrial membrane potential was assessed using JC-1 staining, and reactive oxygen species (ROS) generation was measured using 20,70-dichlorodihydrofluorescein diacetate staining. In addition, the levels of malondialdehyde (MDA) and superoxide dismutase (SOD) levels were also evaluated using the corresponding kits. Flow cytometry was subsequently used to detect apoptosis, and western blotting was used to measure the expression levels of nuclear factor erythroid derived-2-related factor 2 and NADPH quinone oxidoreductase 1. The results showed that high glucose concentration increased oxidative stress and apoptosis in RSC96 cells. Oltipraz improved cell viability and reduced apoptosis of RSC96 cells in the high glucose environment. Additionally, oltipraz exhibited a significant antioxidative effect, as shown by the decrease in MDA levels, increased SOD levels, and reduced ROS generation in RSC96 cells. The results of the present study suggest that oltipraz exhibits potential as an effective drug for treatment with DPN.

## 1. Introduction

Diabetes mellitus (DM) is a systemic metabolic disease characterized by high blood glucose levels. DM is the most common cause of neuropathy worldwide, and up to 50% of all patients with DM may develop neuropathy [1–3]. Diabetic peripheral neuropathy (DPN) is a common complication of DM; ~50% of the patients with DPN are asymptomatic, whereas others may suffer from complicated symptoms such as pain, foot ulcers, and paresthesia [4, 5]. DPN severely affects patients' quality of life and thus presents a significant economic burden to patients.

Schwann cells (SCs) are the most common type of glia in peripheral nerves. SCs ensheath all the axons of the peripheral nerves and secrete neurotrophic factors, which help maintain the structure and function of peripheral nerves [6, 7]. Increasing evidence has shown that SCs serve an important role in DPN [8–13]. SC apoptosis induced by a high glucose environment is thought to be one of the primary causes of DPN. Increased SC apoptosis was observed in diabetic (db/db) mice and diabetic rats treated with streptozotocin [12–15]. A previous study showed that inhibiting SC apoptosis could alleviate myelin sheath injury and delay peripheral nerve degeneration in DPN [15–17].

SCs are hypothesized to be an important site of reactive oxygen species (ROS) generation in peripheral nerves [10]. Hyperglycaemia promotes excessive ROS production in SCs primarily through the polyol (sorbitol) pathway, the mitochondrial electron transport chain, increased production of advanced glycation end products, NADPH oxidases, and nitric oxide synthases [18–20]. Excessive ROS levels induced by hyperglycaemia in SCs can increase intracellular oxidative stress which results in cell apoptosis, promoting the pathological mechanisms underlying peripheral neuropathy [21, 22]. Preventing oxidative stress in SCs may thus be a suitable method for preventing apoptosis of SCs.

Oltipraz [4-methyl-5-(2-pyrazinyl)-1,2-dithiole-3-thione] is an agonist of nuclear factor erythroid derived-2-related factor 2 (Nrf2), an important transcription factor involved in regulating the intracellular antioxidant response [23, 24]. Nrf2 functions as an activator of antioxidant response element (ARE), which is recognized as the cis-element essential for the basal and inducible expression of several antioxidant genes, including NADPH quinone oxidoreductase 1 (NQO-1) [25–27]. Oltipraz and its oxidized metabolites have proven strong antioxidant effects in animal models or clinical patients in certain diseases [28–32]. However, it is still unclear whether oltipraz may reduce apoptosis of SCs through reducing oxidative stress. The aim of the present study was to investigate the effect of oltipraz on apoptosis of SCs induced by high glucose.

## 2. Materials and Methods

### 2.1. Cell Culture and Treatment

RSC96 cells, a rat SC line, was purchased from The Cell Bank of Type Culture Collection of the Chinese Academy of Sciences. RSC96 cells were cultured in DMEM (Gibco; Thermo Fisher Scientific, Inc.) supplemented with 10% FBS (Thermo Fisher Scientific, Inc.) with 5% CO_2_ at 37°C. Cells were trypsinized and subcultured in 6-well plates (5 × 10^5^ cells/well) or 96-well plates (1 × 10^4^ cells/well) for subsequent experiments. After reaching ~70% confluence, RSC96 cells were cultured in DMEM supplemented with 10% FBS (5 mM glucose; control) or high-glucose DMEM supplemented with 10% FBS (50 mM glucose) for 24 h. Cells were pretreated with 20 *μ*M oltipraz (Sigma-Aldrich; Merck KGaA) for 24 h prior to high glucose treatment. For the control conditions, 45 *μ*M mannitol was used to match the hyperosmolality of the cells cultured in the hyperglycemic conditions. The glucose concentrations and oltipraz concentrations used in the present study were based on previous studies [15, 31].

### 2.2. Determination of Cell Viability

The viability of RSC96 cells was assessed using a Cell Counting Kit-8 (CCK-8) assay (KeyGen Biotech). Briefly, cells were subcultured in 96-well plates (1 × 10^4^ cells/well). Following high glucose treatment for 24 h, the cells were incubated with 10 *μ*l CCK-8 solution combined with 100 *μ*l serum-free DMEM at 37°C for 2 h. The absorbance at 450 nm was measured using a microplate reader (Epoch; BioTek Instruments, Inc.).

### 2.3. Determination of Mitochondrial Membrane Potential

JC-1 staining (Beyotime Institute of Biotechnology) was used to evaluate the mitochondrial membrane potential of RSC96 cells. Cells were washed twice with PBS (KeyGen Biotech) and incubated with JC-1 working buffer at 37°C for 20 min. The cells were then washed twice with JC-1 staining buffer and observed under a fluorescence microscope (magnification, x100). In addition, the mitochondrial membrane potential of RSC96 cells was analysed using a BD Accuri C6 Plus flow cytometer (BD Biosciences) and FlowJo software (version 10.0.7). Green fluorescence was detected in the FL1 channel, and red fluorescence was detected in the FL2 channel.

### 2.4. Determination of Cell Apoptosis

RSC96 cell apoptosis was assessed using Annexin V-fluorescein isothiocyanate (FITC)/propidium iodide (PI) staining (BD Biosciences). RSC96 cells were digested with EDTA-free trypsin (Gibco; Thermo Fisher Scientific, Inc.). Cells were washed twice with ice-cold PBS and resuspended in 100 *μ*l binding buffer. Cells were incubated with 5 *μ*l Annexin V-FITC and 5 *μ*l PI for 15 min at room temperature in the dark. Apoptosis was analysed using flow cytometry within 30 min of staining.

### 2.5. Determination of Intracellular ROS

20,70-Dichlorodihydrofluorescein diacetate (DCFH-DA; Sigma-Aldrich; Merck KGaA) was used to assess intracellular ROS levels in RSC96 cells. Cells were collected and resuspended in 10 mM DCFH-DA solution with serum-free DMEM and incubated at 37°C for 20 min. The cells were then washed three times with serum-free DMEM to remove extracellular DCFH-DA. Flow cytometry was used to detect ROS levels with an excitation wavelength of 488 nm and an emission wavelength of 519 nm.

### 2.6. Determination of Malondialdehyde (MDA) and Superoxide Dismutase (SOD) Levels

A lipid peroxidation MDA assay kit (Beyotime Institute of Biotechnology) was used to measure MDA levels, and a WST-8 assay kit (Beyotime Institute of Biotechnology) was used to detect SOD levels according to the manufacturer's protocol. Briefly, cell lysates were incubated with working solution for 30 min at 37°C. Absorbance was measured at 450 nm for SOD and at 523 nm for MDA using a microplate reader. Total protein concentration was measured using a bicinchoninic acid (BCA) protein assay kit (Thermo Fisher Scientific, Inc.) and was used to normalize the MDA and SOD levels.

### 2.7. Western Blot Assay

Western blotting was used to evaluate the expression levels of Nrf2 and NQO1. After 24 h of treatment, total protein was extracted from RSC96 cells using radioimmunoprecipitation assay buffer (Sigma-Aldrich; Merck KGaA). The samples were sonicated for 10 sec. Following centrifugation at 2,000 × g for 15 min at 4°C, protein concentrations were measured using a BCA protein assay kit. Total protein (20 *μ*g per sample) was separated by SDS-PAGE on a 12% gel and subsequently transferred to a nitrocellulose membrane (EMD Millipore). The membranes were incubated overnight at 4°C with the following antibodies: Anti-Nrf2 (1 : 1,000; cat. no. 12721; Cell Signalling Technology Inc.), anti-NQO1 (1 : 1,000; cat. no. ab80588; Abcam), or anti-*β*-actin (1 : 1,000; cat. no. 4970; Cell Signalling Technology, Inc.). Membranes were washed with TSB-Tween (0.05%) for 30 min and incubated with an anti-rabbit secondary antibody (1 : 5,000; cat. no. 7074; Cell Signaling Technology, Inc.) for 2 h at room temperature. Enhanced chemiluminescence Plus (Tanon Science and Technology Co., Ltd.) was used to visualize the protein bands. Densitometry analysis was performed using ImageJ software (version 1.8.0; National Institutes of Health).

### 2.8. Statistical Analysis

Each experiment was independently performed at least three times. Data are expressed as the mean ± standard deviation. Statistical analysis was performed using SPSS software (version 20.0; IBM Corp.). Statistical comparisons among different groups were performed using one-way ANOVA followed by Bonferroni's multiple comparison test. *P* < 0.05 was considered to indicate a statistically significant difference.

## 3. Results

### 3.1. Oltipraz Ameliorates High Glucose Induced RSC96 Cell Injury

**Figure 1:**
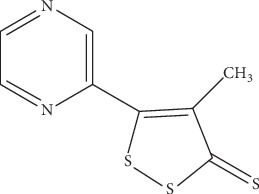
The structural formula of oltipraz.

**Figure 2:**
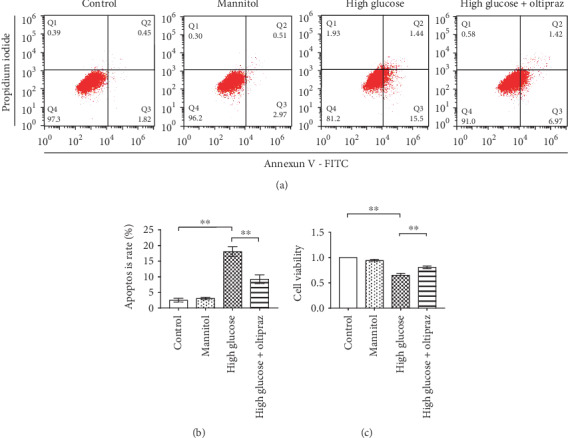
Flow cytometry was used to detect apoptosis of RSC96 cells. (a) Representative flow cytometry histograms. (b) Apoptotic rate of RSC96 cells in the different groups. The apoptotic rate in the high-glucose treatment group was significantly increased compared with the control group. Oltipraz reduced the apoptotic rate compared with high-glucose treatment alone. (c) Cell viability in the different groups after 24 h of treatment. Significant differences were observed among all groups in apoptosis rate and cell viability (*P* < 0.01, one-way ANOVA). ^∗∗^*P* < 0.01.

The structural formula of oltipraz was shown in Figure 1. A CCK8 assay was used to determine the viability of RSC96 cells. The results showed that 50 mM high glucose reduced the viability of RSC96 cells (*P* < 0.01), and treatment with Oltipraz significantly increased cell viability in response to high glucose conditions (*P* < 0.01) (Figure 2(c)).

**Figure 3:**
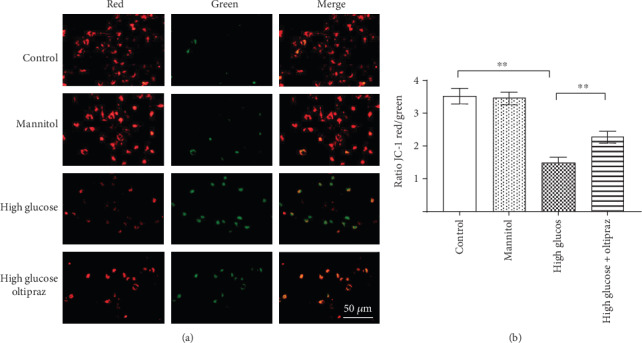
JC-1 staining was used to detect cell apoptosis. (a) Fluorescence microscopy showed that the mitochondrial membrane potential of RSC96 cells was decreased (green fluorescence) in the high-glucose group compared with the control group. Oltipraz prevented mitochondrial damage as a result of treatment with high glucose. (b) Flow cytometry analysis showed that the red/green fluorescence ratio of cells in the high-glucose group was higher compared with the control group. Oltipraz treatment significantly increased the red/green fluorescence ratio compared with the high-glucose group. Scale bar, 50 *μ*m. Significant differences were observed among all groups (*P* < 0.01, one-way ANOVA). ^∗∗^*P* < 0.01.

Depolarized mitochondrial membrane potential is considered a primary marker of early cell apoptosis. JC-1 staining was used to evaluate the mitochondrial membrane potential of RSC96 cells. The control group showed red fluorescence whereas the cells incubated in high-glucose showed increased green fluorescence when observed under the fluorescence microscope, suggesting that high-glucose conditions depolarized the mitochondrial membrane potential in RSC96 cells. Green fluorescence was decreased in the oltipraz-treated cells compared with the cells in the high-glucose group (Figure 3(a)). Flow cytometry analysis also showed that the red/green fluorescence ratio in the oltipraz-treated cells was higher compared with the high-glucose group (*P* < 0.01), suggesting that oltipraz protected RSC96 cells against glucose-induced mitochondrial damage (Figure 3(b)).

Annexin V/PI staining was subsequently used to investigate RSC96 cell apoptosis (Figure 2(a)). Flow cytometry analysis showed that the apoptotic rate in the high-glucose group was significantly increased compared with the control group (*P* < 0.01), and oltipraz treatment significantly reduced apoptosis compared with the high-glucose group (*P* < 0.01), indicating that oltipraz protected against apoptosis induced by high glucose in RSC96 cells (Figure 2(b)). Mannitol was used to normalize the hyperosmolality, and the results showed that hyperosmolality did not affect cell viability or apoptosis.

### 3.2. Oltipraz Inhibits ROS Generation

**Figure 4:**
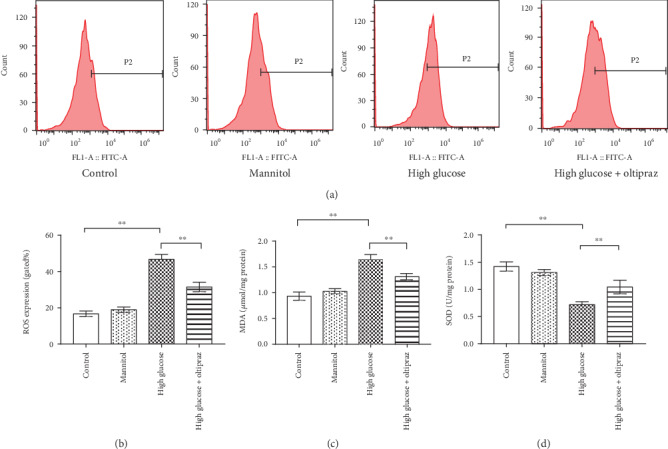
DCFH-DA staining was used to detect RSC96 cell apoptosis. (a) Representative flow cytometry histograms. (b) Flow cytometry analysis showed that high glucose levels significantly increased ROS generation, and oltipraz treatment reduced excessive ROS generation. (c) MDA levels of RSC96 cells in the different groups. (d) SOD levels of RSC96 cells in the different groups. Significant differences were observed among all groups in levels of ROS, MDA, and SOD (*P* < 0.01, one-way ANOVA). ^∗∗^*P* < 0.01. DCFH-DA: 20,70-dichlorodihydrofluorescein diacetate.

After 24 h of treatment, DCFH-DA staining was used to measure the ROS levels of RSC96 cells in different groups (Figure 4(a)). Flow cytometry analysis showed that the proportion of positively stained cells in the high-glucose group was significantly higher compared with the control group (*P* < 0.01). Oltipraz treatment reduced the proportion of positively stain cells in the high glucose-treated cells compared with high-glucose treatment alone (*P* < 0.01). Mannitol did not affect ROS production (Figure 4(b)). The results demonstrated that oltipraz effectively prevented excessive ROS generation as a result of the high glucose conditions.

### 3.3. Oltipraz Increases SOD Levels and Decreases MDA Levels

A lipid peroxidation MDA assay kit was used to measure MDA levels and a WST-8 assay was used to assess SOD levels. The results showed that high glucose conditions increased MDA levels and decreased SOD levels in RSC96 cells (Figures 4(c) and 4(d); *P* < 0.01). Compared with high-glucose treatment alone, oltipraz treatment significantly reduced MDA levels and increased SOD levels (*P* < 0.01). There was no significant difference between the mannitol group and the control group. The lower levels of MDA and higher levels of SOD in the oltipraz-treated cells suggesting that oltipraz protected RSC96 cells from oxidative stress.

### 3.4. Oltipraz Treatment Increases the Expression of Nrf2 and NQO1

**Figure 5:**
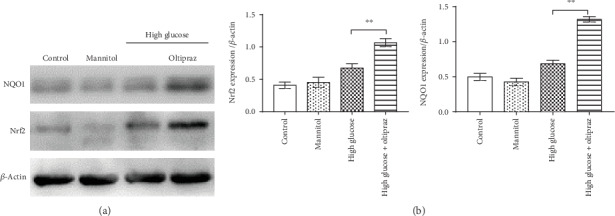
Western blotting was used to assess the expression levels of Nrf2 and NQO1. (a) Representative western blotting image showing Nrf2 and NQO1 expressions in SCs. (b) Oltipraz treatment significantly increased the expression levels of Nrf2 and NQO1 compared with cells incubated in high-glucose conditions without treatment. Significant differences were observed among all groups in Nrf2 and NQO1 expression (*P* < 0.01, one-way ANOVA). ^∗∗^*P* < 0.01. Nrf2: nuclear factor erythroid derived-2-related factor 2; NQO-1: NADPH quinone oxidoreductase 1.

Western blotting was used to measure the expression levels of Nrf2 and NQO1 in RSC96 cells in the four groups. Western blot analysis showed that in the oltipraz groups, the expression levels of Nrf2 and NQO1 were significantly increased compared with the high glucose group (*P* < 0.01). There was no significant difference observed between the mannitol group and the control group (Figure 5). The results show that oltipraz treatment significantly increased the expression of Nrf2 and the downstream molecule NQO1, and thus exerted a potential antioxidant effect.

## 4. Discussion

Previous studies have demonstrated that apoptosis of SCs induced by high glucose contributes to the development of DPN [12–15]. Effective drugs to prevent apoptosis of SCs are important for relieving and reversing peripheral nerve injury caused by DM. The present study showed that oltipraz effectively prevented oxidative stress caused by high glucose, and thus reduced SC apoptosis. Oltipraz treatment decreased MDA levels, increased SOD levels, reversed excessive ROS generation in SCs incubated with high glucose, and protected SCs from mitochondrial damage. Oltipraz treatment increased the expression of Nrf2 and the downstream molecule NQO1 in SCs incubated in a high-glucose environment, and this may underlie the beneficial antioxidant effects of oltipraz.

Peripheral neuropathy is the most common complication of diabetes, with a prevalence of up to 50% in diabetic patients [2–5]. Several drugs have been reported to be less effective, such as oxcarbazepine and acetyl L-carnitine [33–35]. Therefore, we still need to find more effective treatments. Although the pathogenesis of DPN is not fully understood, increasing evidence has shown that SC injury is one of the characteristics of DPN [8–13]. Oxidative stress induced by high glucose conditions in DM is a primary cause of tissue damage. SCs are considered an important producer of ROS. Excessive ROS levels caused by high glucose levels result in mitochondrial damage and apoptosis in SCs, affecting the protective effect and nerve repair function of SCs [9]. In addition, SC interactions with other tissues, including axons and microvessels are disrupted, which contribute to the occurrence and development of DPN [10]. Therefore, preventing oxidative stress in SCs in a high glucose environment, and further preventing the excessive apoptosis of SCs, may be an effective method for treating DPN.

Nrf2 is a transcription factor that controls the basal and induced expression of a range of antioxidant enzymes. The Nrf2 signalling system involves interacting proteins and regulatory molecules to counter oxidative stress. Studies have reported that Nrf2 has an antioxidant effect through inducing expression of the NQO1 isoenzyme [25, 26]. Oltipraz activates Nrf2 and subsequently increases the expression of genes encoding antioxidants. Oltipraz has been shown to be effective in animal models of certain diseases due to its antioxidant properties, including heart failure, acute kidney injury, and liver injury [30–32]. Therefore, oltipraz may potentially inhibit SC apoptosis by preventing oxidative stress.

The present study demonstrated that high glucose-induced oxidative stress promoted RSC96 cell apoptosis. To mimic hyperglycemia, 50 mM glucose was used in the high-glucose group, and 5 mM glucose was used as the control. Mannitol was used to normalize the hyperosmolality of hyperglycemia. The results showed that high-glucose levels promoted ROS generation, increased MDA levels, and decreased SOD levels in SCs, suggesting that high-glucose conditions induced oxidative stress in SCs. Furthermore, incubation with high glucose depolarized the mitochondrial membrane potential, suggesting the presence of mitochondrial damage. Oxidative stress results in SC injury, consistent with the reduced viability and increased apoptosis of SCs treated with high-glucose in the present study. Oltipraz treatment exhibited a strong antioxidant capacity with low ROS levels, low MDA levels, and high SOD levels. Mitochondria are the primary source and target of ROS. Mitochondria are damaged as a result of oxidative stress, and mitochondrial damage is used as a marker of cell apoptosis and oxidative stress [36]. Oltipraz treatment alleviated mitochondrial damage, and thus improved cell viability and prevented cell apoptosis. Western blotting demonstrated that oltipraz increased Nrf2 and NQO1 protein expression in SCs. Thus, it is hypothesized that oltipraz prevented oxidative stress through the activation of the Nrf2/NQO1 signalling pathway.

In conclusion, the present study showed that oltipraz effectively prevents oxidative stress induced by high glucose, and thus reduced SC apoptosis through increasing the expression of Nrf2 and its downstream signalling molecule, NQO1. However, considering that SCs are not the only type of cells damaged in DPN, the effectiveness of oltipraz for the treatment of other types of damaged neural tissues and cells should be examined. In addition, further experiments *in vivo* are required to determine whether oltipraz may effectively relieve the symptoms of DPN. However, the present study highlights the potential of oltipraz for the treatment of DPN.

## Data Availability

The data used to support the findings of this study are available from the corresponding author upon request.

## Abbreviations

DM: Diabetes mellitus
DPN: Diabetic peripheral neuropathy
SC: Schwann cell
ROS: Reactive oxygen species
MAPK: Mitogen-activated protein kinase
Nrf2: Nuclear factor erythroid derived-2-related factor 2
NQO-1: NADPH quinone oxidoreductase 1.
